# Supplementary material for: Investigation of Vestibular Function in Adult Patients with Gitelman Syndrome: Results of an Observational Study
Source: J Clin Med. 2020 Nov 23;9(11):3790. doi: 10.3390/jcm9113790 (PMC7700665; doi:10.3390/jcm9113790)
Supplement: Supplementary file 1 [file jcm-09-03790-s001.pdf]

SUPPLEMENTAL MATERIAL TABLE OF CONTENTS

- Table S1. Responses of the Gitelman patients to the survey on vertigo clinical presentation.
- Table S2. Variants detected in Gitelman syndrome patients in the *SLC12A3* gene and their classification.
- Table S3. Baseline characteristics and treatment of the sixteen Gitelman syndrome patients with vertigo.
- Table S4. Comparative biological and clinical results between patients with normal and abnormal vestibular function.
- Figure S1. Ion exchanges in the endolymphatic sac.
- Figure S2. Clinical set up and principle of the Video Head Impulse Test (VHIT).
- Figure S3. Normal result to Video Head Impulse Test (VHIT).
- Figure S4. Clinical set up and principle of kinetic tests.
- Figure S5. Clinical set up and principle of caloric tests.
- Supplemental material references.

Table S1. Responses of the Gitelman patients to the survey on vertigo clinical presentation.

	Number of patients (%)
**What do you mean by "vertigo"?**	n=16
- Do you feel dizzy?	9 (56)
- Do you feel drunk?	5 (31)
- Do you lose equilibrium?	8 (50)
- Do objects rotate in one direction?	4 (25)
- Do objects turn in several directions?	2 (12)
**What is the duration?**	n=15
- Less than 10 minutes	13 (87)
- Between 10 minutes and one hour	0 (0)
- Several hours/days	2 (13)
- Almost permanent	0 (0)
**Are there associated signs?**	n=16
- Moderate fatigue	5 (31)
- Severe fatigue	4 (25)
- Nausea	5 (31)
- Headache	2 (12)
- Tinnitus	6 (37)
**What triggers vertigo crises?**	n=16
- The transition from lying to standing position and reverse	7 (44)
- Rotation of the head or to look up or down	9 (56)
- Turning in the bed	1 (6)
- No apparent trigger	4 (25)
**Who did you consult?**	n=16
- An ear, nose, and throat specialist	2 (12)
- Your general practitioner	4 (25)
- Your nephrologist	10 (63)
- No physician	0 (0)
**Do you consider vertigo as a limitation to your daily life?**	n=14
- No	4 (29)
- A little	8 (57)
- A lot (i.e., it limits important aspect of daily life)	2 (14)
**Do you consider vertigo as a limitation to your work?**	n=14
- No	7 (50)
- A little	5 (36)
- A lot	1 (7)
- It prevents me from working	1 (7)

Table S2. Variants in the *SLC12A3* gene detected in Gitelman syndrome patients in the cohort and their classification. Variants were classified according the ACMG recommendations, which are based on several criteria including population data, computational data, functional data, and segregation data [1], into five categories: class 1, benign; class 2, likely benign; class 3, uncertain significance; class 4, likely pathogenic; and class 5, pathogenic.

Patient	Allele 1				Allele 2			
	cDNA	protein	ACMG class	Ref	cDNA*	protein	ACMG class	Ref
1	c.1946C>T	p.Thr649Met	4	[2]	c.(?_-30)_(964+1_965-1) (E1_E7del)	p.?	5	[3,4]
2	c.1456G>A	p.Asp486Asn	4	[5]	c.1924C>G	p.Arg642Gly	5	[6]
3	c.2221G>A	p.Gly741Arg	5	[5,7,8]	c.2981G>A	p.Cys994Tyr	5	[8,9]
4	c.1967C>T	p.Pro656Leu	4	[3]	c.2191G>A	p.Gly731Arg	5	[4,10]
5	c.56_57dup	p.Phe20Alafs*8	5	[11]	c.56_57dup	p.Phe20Alafs*8	5	[11]
6	c.2660+1G>A	p.?	5	[12]	c.2660+1G>A	p.?	5	[12]
7	c.2576T>C	p.Leu859Pro	5	[5,10]	c.3052C>T	p.Arg1018*	5	[13]
8	c.2576T>C	p.Leu859Pro	5	[5,10]	c.3052C>T	p.Arg1018*	5	[13]
9	c.3077C>T	p.Thr1026Ile	5	[7]	c.3077C>T	p.Thr1026Ile	5	[7]
10	c.2191G>A	p.Gly731Arg	5	[4,10]	c.2576T>C	p.Leu859Pro	5	[5,10]
11	c.1967C>T	p.Pro656Leu	4	[3]	c.2191G>A	p.Gly731Arg	5	[4,10]
12	c.1519C>T	p.Arg507Cys	4	[3]	c.2981G>A	p.Cys994Tyr	5	[8,9]
13	c.1925+1G>A	p.?	5	[2]	c.1001G>A	p.Arg334Gln	4	This study
14	c.2576T>C	p.Leu859Pro	5	[5,10]	c.2581C>T	p.Arg861Cys	5	[14]
15	c.2089_2095del	p.Thr697Glyfs*2	5	[9]	c.2379dup	p.Phe794Valfs*2	5	[2]
16	c.2883+1G>T	p.?	5	[5]	c.2883+1G>T	p.?	5	[5]

Table S3. Baseline characteristics and treatment of the sixteen Gitelman syndrome patients with vertigo.

	**Sex**	**Age at diagnosis**	**KCl supplementation**	**Mg supplementation**	**Other treatment**
**1**	M	12 yrs	6 g	100 mg	indomethacin 75 mg
**2**	F	29 yrs	5 g	100 mg	-
**3**	M	25 yrs	7 g	-	-
**4**	F	34 yrs	2 g	100 mg	-
**5**	M	34 yrs	2 g	100 mg	-
**6**	F	32 yrs	9 g	200 mg	spironolactone 25 mg
**7**	F	10 yrs	4 g	400 mg	-
**8**	M	6 yrs	6 g	200 mg	-
**9**	F	32 yrs	4 g	200 mg	-
**10**	F	18 months	5 g	600 mg	-
**11**	F	31 yrs	1 g	-	-
**12**	F	32 yrs	3,5 g	200 mg	spironolactone 25 mg
**13**	F	20 yrs	9 g	700 mg	spironolactone 25 mg
**14**	F	42 yrs	6 g	100 mg	-
**15**	M	38 yrs	3 g	200 mg	-
**16**	M	10 yrs	8 g	-	spironolactone 50 mg

Table S4. Comparative biological and clinical results between patients with normal and abnormal vestibular function.

	**Abnormal vestibular function (n=7)**	**Normal vestibular function (n=9)**	**p-value**
Plasma K^+, mmol/L	3.2 [2.8; 3.35]	2.6 [2.2; 2.9]	0.17
Plasma Mg^{2+}, mmol/L	0.60 [0.53; 0.7]	0.59 [0.54; 0.64]	0.96
SBP sitting, mmHg	117 [111; 125]	114 [108; 118]	0.52
SBP standing, mmHg	110 [106; 124]	109 [100; 119]	0.52
DBP sitting, mmHg	72 [68; 78]	70 [66; 76]	0.92
DBP standing, mmHg	76 [73; 78]	74 [73; 79]	0.96
HR sitting, bpm	86 [82; 104]	78 [74; 81]	0.05
HR standing, bpm	99 [87; 114]	90 [86; 94]	0.31
Chondrocalcinosis, n	3	5	1.00
Hearing loss, n	3	3	1.00

SBP: systolic blood pressure; DBP: diastolic blood pressure; HR: heart rate; bpm: beats per minute. For quantitative values, results are expressed as medians [interquartile range].

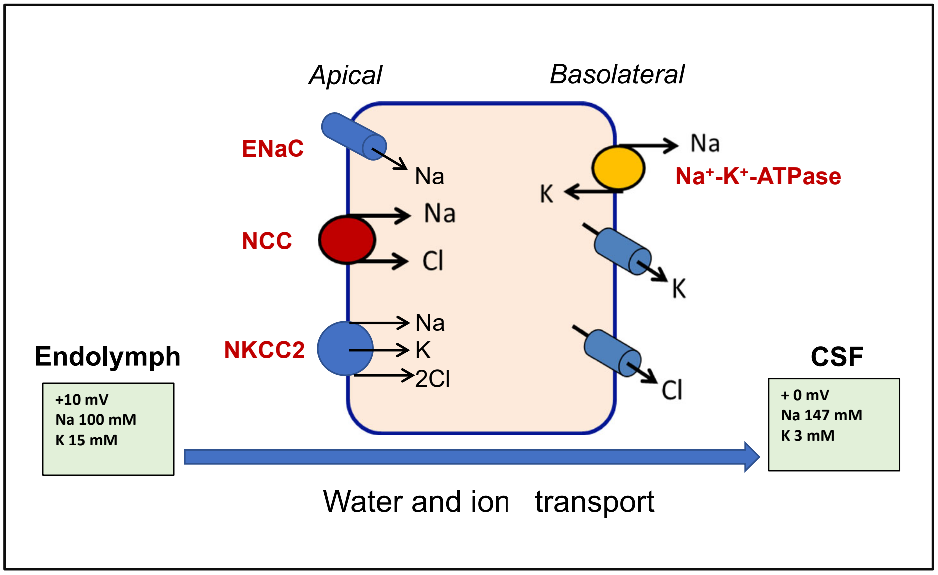

Figure S1. Ion exchanges in the endolymphatic sac. The endolymphatic sac regulates endolymph homeostasis through control of ion and water transport from epithelial cells of the endolymphatic sac. The molecules involved include ion channels such as the epithelial sodium channel ENaC, localized at the apical membrane, and K^+ and Cl^- channels, localized at the basolateral membrane; cotransporters such as thiazide-sensitive Na^+-Cl^- cotransporter NCC and bumetanide-sensitive Na^+-K^+-$2Cl^-$ cotransporter 2 (NKCC2). An Na^+,K^+-ATPase at the basolateral membrane is also involved. Figure adapted from Mori et al. [15]. CSF: cerebrospinal fluid.

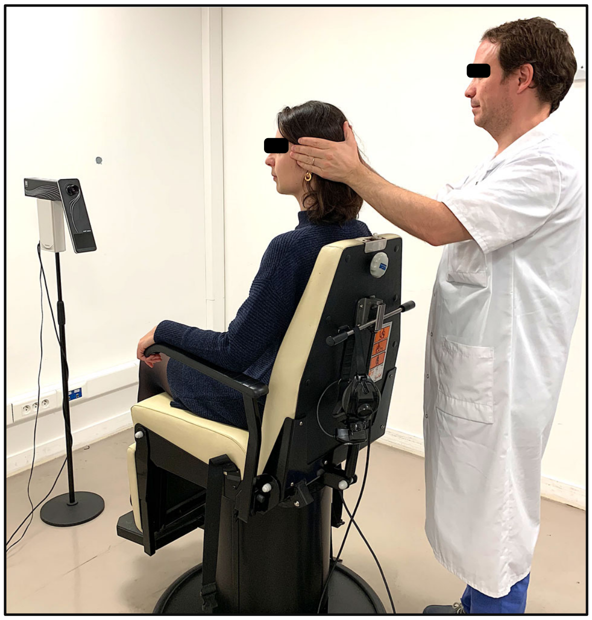

Figure S2. Clinical set up and principle of the Video Head Impulse Test (VHIT). The clinician stands behind the patient and abruptly moves the patient's head in the plane of each semicircular canal (SCC) while the patient looks at a lighted, fixed target located 90 cm in front of him or her. The clinician moves the patient's head in six different directions corresponding to the orientation of stimulation of each SCC. The camera tracks eye movements and registers the head movement simultaneously. The system focuses on the ability to keep eyes fixed on the target, which is detected by an absence of movement of the eyes while the head is moving. If there is a vestibular abnormality, the eyes do not remain focused but instead move with the head and then make a saccade (quick backward movement) to return to the target [16].

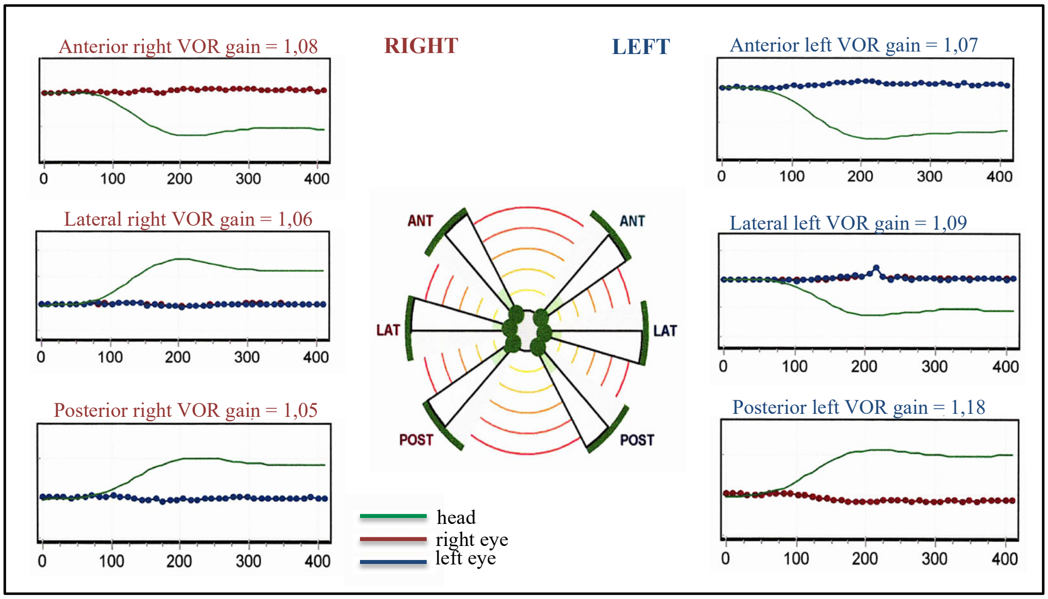

Figure S3. Normal result in the Video Head Impulse Test (VHIT). Each semi-circular canal is tested during this test. Lateral panels represent from top to bottom: anterior (ANT), lateral (LAT), and posterior (POST) semicircular canal (SCC). Left panels represent the right SCC, and the right panels represent the left SCC. Green lines correspond to head movements; blue dotted lines correspond to the left eye movements; and red dotted lines correspond to the right eye movements. In this normal result, the eyes stayed focused on the target (no movement) while the head moved. The central figure represents the sum of responses for each SCC. The green dots are located in the light green portion of the figure circle, showing that all responses were normal during the test and that the vestibulo-ocular reflex gain was normal (between 0.8 and 1.2). If the test was abnormal, the dots would be colored in red and would be located outside the light green portion.

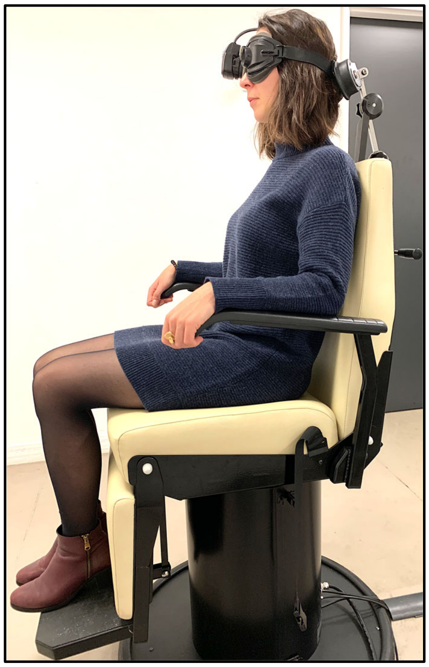

Figure S4. Clinical set up and principle of kinetic tests. The patient is seated on a rotatory chair wearing a mask. The chair moves in a sinusoidal way for the sinusoidal harmonic acceleration test, whereas it makes a quick acceleration followed by a quick deceleration in the impulsive rotational test [17]. Normally, the head stays fixed with the rest of the body during the test. In other words, the head and body move with the chair as one single element. These movements of the chair generate a vestibular stimulation that is manifested by a nystagmus. We used videonystagmoscopy (an infrared camera located in the mask, which films eye movements) to observe and quantify the intensity and the frequency of the nystagmus. If the result is normal, the velocity of the eyes are symmetrical [18]. The test result is considered abnormal if there is a directional preponderance (left or right eye) of 2°/second or more.

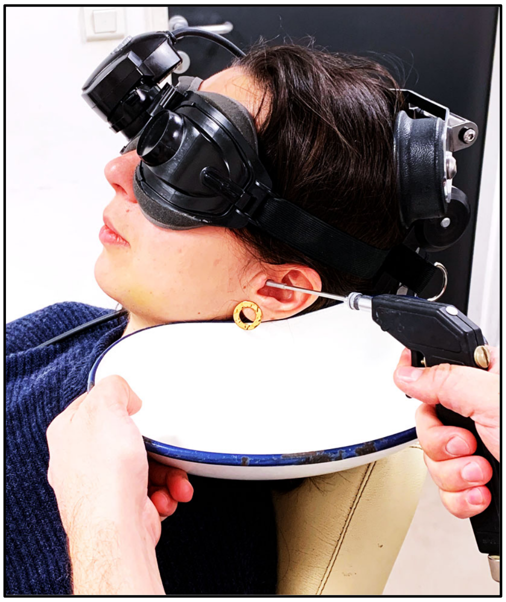

Figure S5. Clinical set up and principle of caloric tests. The patient wears a mask and is seated with the chair back at an angle 30° from the vertical plan. The lateral semi-circular canal is the only canal tested. The ears are alternatively irrigated for 30 seconds with 200 mL of cold (30 °C) then hot (44 °C) water. This irrigation creates a nystagmus. A hot-water irrigation induces an excitation of the vestibule generating a nystagmus beating toward the stimulated ear, whereas a cold-water irrigation induces an inhibition of the vestibule generating a nystagmus beating toward the non-stimulated ear. The induced nystagmus is recorded with videonystagmoscopy until it stops. Lateral canal dysfunction is defined as a difference of 15% or more between the responses of the two ears [18] .

Supplemental material References

1. Richards S.; Aziz N.; Bale S.; Bick D.; Das S.; Gastier-Foster J.; Grody W.W.; Hedge M.; Lyon E.; Spector E.; Voelkerdin K.; Rehm H.L. Standards and guidelines for the interpretation of sequence variants: a joint consensus recommendation of the American College of Medical Genetics and Genomics and the Association for Molecular Pathology. *Off J Am Coll Med Genet* **2015**, 17(5):405–24.
2. Ji W.; Foo J.N.; O'Roak B.J.; Zhao H.; Larson M.G.; Simon D.B.; Newto-Cheh C.; State M.W.; Levy D.; Lifton R.P. Rare independent mutations in renal salt handling genes contribute to blood pressure variation. *Nat Genet* **2008**, 40(5):592–9.
3. Vargas-Poussou R.; Dahan K.; Kahila D.; Venisse A.; Riveira-Munoz E.; Debaix H.; Grisart B.; Bridoux F.; Unwin R.; Moulin B.; Haymann J.P.; Vantyghem M.C.; Rigothier C.; Dussol B.; Godin M.; Nivet H.; Dubourg L.; Tack I.; Gimenez-Roqueplo A.P.; Houillier P.; Blanchard A.; Devuyst O.; Jeunemaitre X. Spectrum of mutations in Gitelman syndrome. *J Am Soc Nephrol* **2011**, 22(4):693–703.
4. Mastroianni N.; Bettinelli A.; Bianchetti M.; Colussi G.; De Fusco M.; Sereni F.; Ballabio A.; Casari G. Novel molecular variants of the Na-Cl cotransporter gene are responsible for Gitelman syndrome. *Am J Hum Genet* **1996**, 59(5):1019–26.
5. Simon D.B.; Nelson-Williams C.; Bia M.J.; Ellison D.; Karet F.E.; Molina A.M.; Vaara I.; Iwata F.; Cushner H.M.; Koolen M.; Gainza F.J.; Gitleman H.J.; Lifton R.P. Gitelman's variant of Bartter's syndrome, inherited hypokalaemic alkalosis, is caused by mutations in the thiazide-sensitive Na-Cl cotransporter. *Nat Genet* **1996**, 12(1):24–30.
6. Cruz D.N.; Simon D.B.; Nelson-Williams C.; Farhi A.; Finberg K.; Burleson L.; Gill J.R.; Lifton R.P. Mutations in the Na-Cl cotransporter reduce blood pressure in humans. *Hypertens* **2001**, 37(6):1458–64.
7. Kunchaparty S.; Palcso M.; Berkman J.; Velázquez H.; Desir G.V.; Bernstein P.; Reilly R.F.; Ellison D.H. Defective processing and expression of thiazide-sensitive Na-Cl cotransporter as a cause of Gitelman's syndrome. *Am J Physiol* **1999**, 277(4):F643-649.
8. Syrén M.L.; Tedeschi S.; Cesareo L.; Bellantuono R.; Colussi G.; Procaccio M.; Ali A.; Domenici R.; Malberti F.; Sprocati M.; Sacco M.; Miglietti N.; Edefonti A.; Sereni F.; Casari G.; Coviello D.A.; Bettinelli A. Identification of fifteen novel mutations in the SLC12A3 gene encoding the Na-Cl Co-transporter in Italian patients with Gitelman syndrome. *Hum Mutat* **2002**, 20(1):78.
9. De Jong J.C.; Van Der Vliet W.A.; Van Den Heuvel L.P.W.J.; Willems P.H.G.M.; Knoers N.V.A.M.; Bindels R.J.M. Functional expression of mutations in the human NaCl cotransporter: evidence for impaired routing mechanisms in Gitelman's syndrome. *J Am Soc Nephrol* **2002**, 13(6):1442–8.
10. Valdez-Flores M.A.; Vargas-Poussou R.; Verkaart S.; Tutakhel O.A.Z.; Valdez-Ortiz A.; Blanchard A.; Treard C.; Hoenderop J.G.J.; Bindels R.J.M.; Jelen S. Functionomics of NCC mutations in Gitelman syndrome using a novel mammalian cell-based activity assay. *Am J Physiol Renal Physiol* **2016**, 311(6):F1159–67.
11. Blanchard A.; Vallet M.; Dubourg L.; Hureaux M.; Allard J.; Haymann J.P.; De la Faille R.; Arnoux A.; Dinut A.; Bergerot D.; Becker P.H.; Courand P.Y.; Baron S.; Houillier P.; Tack I.; Devuyst O.; Jeunemaitre X.; Azizi M.; Vargas-Poussou R. Resistance to Insulin in Patients with Gitelman Syndrome and a Subtle Intermediate Phenotype in Heterozygous Carriers: A Cross-Sectional Study. *J Am Soc Nephrol* **2019**.
12. Fava C.; Montagnana M.; Rosberg L.; Burri P.; Almgren P.; Jönsson A.; Wanby P.; Lippi G.; Minuz P.; Hulthen L.U.; Aurell M.; Melander O. Subjects heterozygous for genetic loss of function of the thiazide-sensitive cotransporter have reduced blood pressure. *Hum Mol Genet* **2008**, 17(3):413–8.
13. Fukuyama S.; Okudaira S.; Yamazato S.; Yamazato M.; Ohta T. Analysis of renal tubular electrolyte transporter genes in seven patients with hypokalemic metabolic alkalosis. *Kidney Int* **2003**, 64(3):808–16.
14. Lemmink H.H.; Knoers N.V.; Károlyi L.; van Dijk H.; Niaudet P.; Antignac C.; Guay-Woodford L.M.; Goodyer P.R.; Carel J.C.; Hermes An Seyberth H.W.; Monnens L.A.; Van den Heuvel L.P. Novel mutations in the thiazide-sensitive NaCl cotransporter gene in patients with Gitelman syndrome with predominant localization to the C-terminal domain. *Kidney Int* **1998**, 54(3):720–30.
15. Mori N.; Miyashita T.; Inamoto R.; Matsubara A.; Mori T.; Akiyama K.; Hoshikawa H. Ion

transport its regulation in the endolymphatic sac: suggestions for clinical aspects of Meniere's disease. *Eur Arch Otorhinolaryngol* **2017**, 274(4):1813–20.
16. Halmagyi G.M.; Chen L.; MacDougall H.G.; Weber K.P.; McGarvie L.A.; Curthoys I.S. The Video Head Impulse Test. *Front Neurol* **2017**, 8:258.
17. Eza-Nuñez P.; Fariñas-Alvarez C.; Fernandez N.P. Comparison of three diagnostic tests in detecting vestibular deficit in patients with peripheral vestibulopathy. *J Laryngol Otol* **2016**, 130(2):145–50.
18. Bhansali S.A.; Honrubia V. Current status of electronystagmography testing. *Off J Am Acad Otolaryngol-Head Neck Surg* **1999**, 120(3):419–26.
